# Antitumor-Directed Fractionation of *Lophocereus marginatus* Extracts Against Murine L5178Y-R Lymphoma Cells

**DOI:** 10.3390/ph19030369

**Published:** 2026-02-26

**Authors:** Ángel David Torres-Hernández, César Iván Romo-Sáenz, Ramiro Quintanilla-Licea, Diana Elia Caballero-Hernández, Jesica María Ramírez-Villalobos, Diana Laura Clark-Pérez, Celia María Quiñonez-Flores, Joel Horacio Elizondo-Luevano, Patricia Tamez-Guerra, Ricardo Gomez-Flores

**Affiliations:** 1 Laboratory of Immunology and Virology, Faculty of Biological Sciences, Universidad Autónoma de Nuevo León, San Nicolás de los Garza 66455, Nuevo León, Mexico; angel.torreshr@uanl.edu.mx (Á.D.T.-H.); diana.caballerohr@uanl.edu.mx (D.E.C.-H.); jessica.ramirezvll@uanl.edu.mx (J.M.R.-V.); diana.clarkp@uanl.edu.mx (D.L.C.-P.); joel.elizondolv@uanl.edu.mx (J.H.E.-L.); patricia.tamezgr@uanl.edu.mx (P.T.-G.); 2 Laboratory of Phytochemistry, Department of Chemistry, Faculty of Biological Sciences, Universidad Autónoma de Nuevo León, San Nicolás de los Garza 66455, Nuevo León, Mexico; ramiro.quintanillalc@uanl.edu.mx; 3 School of Medicine and Biomedical Sciences, Universidad Autónoma de Chihuahua, Chihuahua 31109, Chihuahua, Mexico; cquinonezf@uach.mx

**Keywords:** Ethnobotany, Mexican cactus, *Lophocereus marginatus*, plant phytochemicals, phenolic compounds, anti-cancer effect, antioxidant effect, mice toxicity

## Abstract

**Background/Objectives**: Cancer has been associated with significant morbidity and mortality worldwide, particularly related to chemotherapy resistance. Therefore, it is essential to investigate alternative sources of non-toxic antitumor compounds. The cactus *Lophocereus marginatus* is native to Mexico and is commonly used to treat gastrointestinal infections and diabetes in traditional medicine. **Methods**: The in vitro antitumor activity of *L. marginatus* extract fractions against murine L5178Y-R lymphoma cells was evaluated. The crude extract and its solvent-derived fractions were evaluated for cytotoxicity, selectivity, and hemolytic activity. **Results**: The crude extract exhibited an IC_50_ of 9.09 μg/mL, demonstrating a high selectivity index (SI: 330.03), with no hemolytic activity observed at 1000 μg/mL. The LM-HP, LM-CP, and LM-MP partitions showed varying IC_50_ values (6.74, 7.93, and 45.38 μg/mL, respectively) and selectivity indices of 445.1, 378.31, and 66.1, respectively. Only LM-HP induced hemolysis at 200 μg/mL. The most promising fraction, CP-F8, exhibited an IC_50_ of 11.2 μg/mL, high selectivity index (354.29), and antioxidant activity, without hemolytic effects. Phytochemical analysis of CP-F8 identified phenolic compounds, triterpenes, and sterols, which are known for their anti-cancer and anti-inflammatory properties. In vivo tests showed no significant liver damage or changes in body weight, indicating the safety of CP-F8. **Conclusions**: These results suggest that CP-F8 is a promising antitumor candidate with selective cytotoxicity and minimal toxicity to normal cells.

## 1. Introduction

Cancer is a group of diseases that affect various organs due to the rapid and uncontrolled growth of abnormal cells [1]. Currently, the Mexican states of Nuevo León, Sonora, Sinaloa, and Chihuahua have the highest rates of cancer in Mexico, with 75 deaths per 100,000 inhabitants [2]. First-line treatments such as surgery, radiotherapy, and chemotherapy may be ineffective and trigger adverse reactions such as systemic toxicity, which affects the patient’s quality of life and cellular resistance [3]. Throughout history, plants have served as an alternative treatment for various human ailments and have become a source of new compounds, constituting a large part of current pharmaceutical agents in the field of cancer therapy [4,5]. *Lophocereus marginatus*, commonly known as organ pipe cactus, is widely distributed in Mexico [6] and has traditionally been used in infusions or raw to treat gastrointestinal ailments, as it has anti-diarrheal and hypoglycemic effects, which is why it is considered an antidiabetic [7]. Its anti-cancer activity has recently been described in in vitro and in vivo studies, which can be attributed to the presence of lophenol, β-sitosterol, and palmitic acid isolated from low polarity extracts [8,9,10]. However, the medium polarity extracts have not yet been investigated, thus in this study we characterized possible metabolites and determined their safety and anti-cancer properties.

## 2. Results

**Figure 1:**
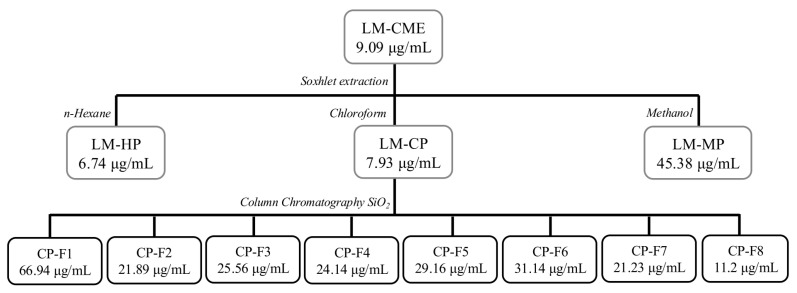
Bio-directed fractionation of *L. marginatus.* IC_50_ represents inhibitory activity against L5178Y-R cells. LM-CME: *L. marginatus* crude methanol extract; LM-HP: *L. marginatus n*-hexane partition; LM-CP: *L. marginatus* chloroform partition; LM-MP: *L. marginatus* methanol partition; and CP-F: chloroform partition fraction.

The general experimental protocol for the extraction and fractionation of *L. marginatus* crude methanol extract is summarized in Figure 1, showing the extraction method and the IC_50_ obtained against L5178Y-R cells.

### 2.1. Biological Activity of L. marginatus Crude Extract and Partitions

The extraction yield of *L. marginatus* and subsequent partitions are presented in Table 1. The initial inhibitory activity of the crude methanol extract (LM-CME) against L5178Y-R cells was 9.09 μg/mL, calculating an activity against PBMCs greater than 3000 μg/mL, with a selectivity index (SI) of 330.03. It is considered non-hemolytic activity when the IC_50_ value is higher than 1000 μg/mL. Obtaining the partitions with solvents of increasing polarity (*n*-hexane, chloroform, and methanol) allowed us to determine that the hexane partition (LM-HP) possessed the highest antitumor activity with an IC_50_ of 6.74 μg/mL against L5178Y-R cells and an SI of 445.1. However, it induced hemolysis at an IC_50_ < 200 μg/mL. The chloroform partition (LM-CP) presented the second best activity of 7.93 μg/mL against lymphoma cells and an SI of 378.31, with hemolytic activity at a concentration > 1000 μg/mL. Furthermore, the methanol partition (LM-PM) had the lowest activity against lymphoma cells with an IC_50_ of 45.38, an SI of 66.1, and an hemolityic activity > 1000 μg/mL. For the three partitions, we observed an hemolitiyc activity > 3000 μg/mL against PBMC.

### 2.2. Column Fractionation of LM-CP

**Figure 2:**
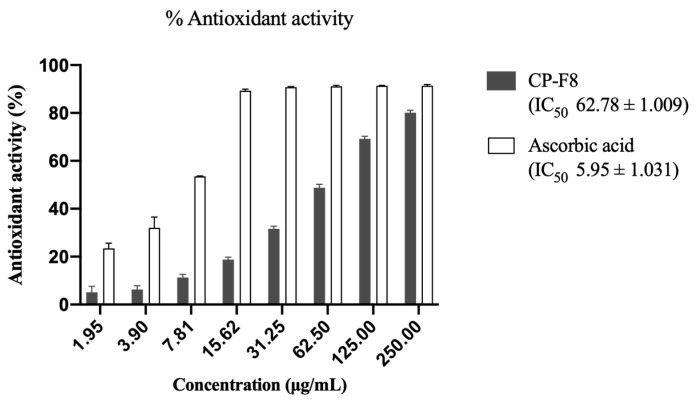
Antioxidant activity percentages of CP-F8 fraction. IC_50_ values represent the mean ± SEM of triplicate determinations from three independent experiments.

Eight LM-CP fractions were obtained, showing yields from 1.26% to 18.05%. Yields and biological activity are shown in Table 2. Fraction CP-F8 had the highest yield and showed the best activity against L5178Y-R cells, with an IC_50_ of 11.2 μg/mL and an SI of 354.29. Due to CP-F8 yield, biological activity, and selectivity, it was further evaluated for lack of toxicity. It was determined that CP-F8 did not induce hemolysis, with an IC_50_ > 1000 μg/mL and an antioxidant activity of 62.78 μg/mL (Figure 2).

### 2.3. Phytochemical Evaluation of CP-F8

The phytochemical profile of CP-F8 was determined in a single experiment using qualitative tests in which physical changes, mainly color changes, were visually evaluated as described in Table 3. The results were interpreted as positive for the presence of carbon-carbon unsaturations, phenolic hydroxyl groups, and steroid and triterpene metabolites in the CP-F8 fraction.

### 2.4. Maximum Tolerated Dose of the CP-F8 Fraction in a Murine Model

**Figure 3:**
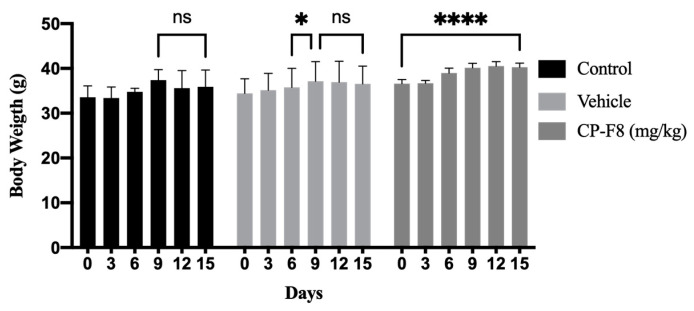
Body weight of mice treated with CP-F8 fraction, vehicle and control untreated for 14 d. ns: not significant, * *p* < 0.05 and **** *p* < 0.01.

In vivo studies were developed to determine the safety of CP-F8, establishing the maximum tolerated dose using concentrations of the fraction at 100 mg/kg and 250 mg/kg of mouse weight, dissolved in the vehicle. At 250 mg/kg, mice did not survive for more than one hour after administration. However, mice treated with the vehicle or 100 mg/kg CP-F8 showed 100% survival at 15 d of treatment. We did not observe weight loss in the groups treated with the extract and the vehicle (Figure 3).

The liver function test performed for each group revealed differences in values between the groups (Table 4), which presented parameters outside the reported reference values for alanine aminotransferase (ALT) activity in the control and vehicle groups. The group treated with the CP-F8 fraction only presented an alkaline phosphatase (ALP) function outside the reported reference values.

**Figure 4:**
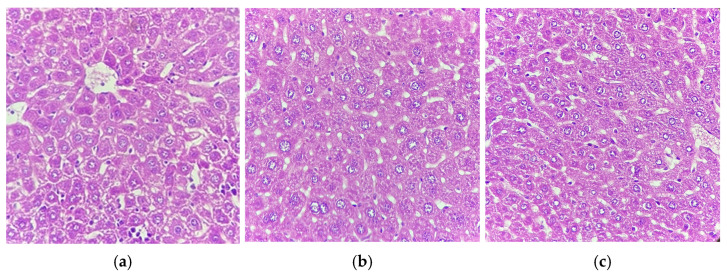
Histological analysis of a Balb/c mouse liver. Image observed at 40×. Image (**a**): histological section of specimen corresponding to the control group; (**b**): corresponding to the vehicle group; (**c**): corresponding to the group treated with 100 mg/kg CP-F8.

Histological analysis of mouse livers from a representative sample was performed on the treated, vehicle, and control groups (Figure 4). Hematoxylin and eosin staining eliminate any type of liver dysfunction, as no signs of hepatocellular damage, necrosis, or fibrosis were observed.

## 3. Discussion

In botanical medicine, cacti have been traditionally used for their anti-inflammatory and anti-cancer properties [12]. In this study, the potential cytotoxic effect of the cactus *L. marginatus* against the L5178Y-R tumor cell line was evaluated, as well as the selectivity and safety of the crude extract and partitions obtained with low, medium, and high polarity solvents (Table 1). Initially, the crude extract showed an IC_50_ of 9.09 μg/mL, a selectivity of 330.03, and was not hemolytic at a concentration of 1000 μg/mL. These cytotoxicity results are consistent with previous studies. However, its selectivity using PBMC and its hemolytic activity on human erythrocytes has not yet been reported [8,9]. IC_50_ of 6.74, 7.93, and 45.38 μg/mL were determined for LM-HP, LM-CP, and LM-MP partitions, respectively. The selectivity of the extracts against cancer is fundamental when investigating alternatives for treatment since the aim is to avoid toxicity in healthy cells at the same concentrations as those in therapy [13]. In this case, LM-HP, LM-CP, and LM-MP showed SIs of 445.1, 378.31, and 66.1 respectively, being considered highly selective against lymphoma, since an SI value greater than two is considered non-toxic for normal cells [14].

In addition, the hemolysis assay allowed us to complement the in vitro toxicity assessment of the extracts [15,16], in which it was observed that the LM-HP partition produced hemolysis at 200 μg/mL, as compared with that of LM-CP and LM-MP fractions, which did not present hemolytic activity, with an IC_50_ > 1000 μg/mL. It has been reported that plant extracts obtained with *n*-hexane exhibit hemolytic activity due to the presence of nonpolar fatty acid compounds [17]. In addition, it was shown that oleanolic acid, as an aglycone in saponins, confers significant hemolytic activity compared with other fatty acids [18]. This hemolytic effect is proportional to the number of carbon atoms present in unsaturated fatty acids, with an increase in activity observed in compounds containing more than 12 carbon atoms in their structure [19], which may be related to palmitic acid, a 16-carbon fatty acid that was previously isolated in a hexane fraction of *L. marginatus* [10]. These compounds primarily interact with the cell membrane through the initial insertion of the fatty acid into the erythrocyte’s lipid bilayer, compromising its integrity and facilitating the entry of more fatty acids in a second penetration [17,20].

According to Canga et al. [21], extracts showing IC_50_ values < 30 μg/mL may be candidates for further antitumor study. Since the LM-CP fraction showed the best selectivity, safety, and biological activity, fractionation was continued using column chromatography, yielding eight fractions with IC_50_ activity ranging from 11.20 μg/mL to 66.94 μg/mL. However, some of these fractions showed low yields or low selectivity (Table 2), thus CP-F8 was selected, which obtained the best yield, an IC_50_ of 11.2 μg/mL, an SI of 354.29, and an antioxidant activity of 62.78 μg/mL, and did not produce hemolysis at 1000 μg/mL.

Qualitative phytochemical analysis of CP-F8 identified metabolites containing unsaturated carbon–carbon (C=C) bonds, phenols, triterpenes, and sterols (Table 3). Simple phenolics are considered to be those containing a substituted phenol unit. However, hydroxycinnamic acid, a class of phenolic acids, also has an unsaturated C=C bond in its structure [22]. This type of compound possesses significant antioxidant activity due to its potential to donate hydrogen atoms and neutralize free radicals, key to its pharmacological property to function as an anti-inflammatory and anti-cancer agent [23]. On the other hand, plant-derived steroids have a structure comprising four cycloalkane rings and various functional groups, mainly hydroxyl groups and a substituent at C17, which provide them with their anti-inflammatory and antitumor properties [24].

Previous studies on *L. marginatus* reported the presence of the phytosteroids lophenol and β-sitosterol in a hexane extract [10]. However, there are currently no reports of this in extracts with an intermediate polarity. In other species of the genus *Lophocereus*, such as *L. schottii*, the presence of polyphenolic compounds such as α-tocotrienol, phenolic acids such as coumaryl and malic acid, as well as steroid compounds such as scoteonol and poriferasta-trienol, has been reported in a polar fraction [25]. Compounds such as phenolic acids, tocotrienols, and phytosteroids have been reported to have anti-cancer activity against diverse types of cancer, including colon, liver, prostate, lung, breast, pancreatic, and leukemia, by inducing apoptosis-type cell death. Therefore, it is suggested that this class of compounds may be present in the fraction, which confers the observed cytotoxic activity [26,27,28,29].

In preclinical studies, a decrease or alteration in body weight is indicative of external stress directly affecting the mouse and its food intake [30]. However, in our study we observed a significant increase in mouse body weights during the 15 days following the application of CP-F8 (Figure 3). Monitoring of liver function to determine anatomical or biochemical alterations is performed by analyzing the enzymes ALT, aspartate aminotransferase (AST), and ALP [31]. Several authors define drug-induced hepatocellular injury as a combined increase in the enzymes ALT, AST, and ALP, mainly observing AST and ALT > 5-fold increases in the upper limit of the normal range, whereas a >2-fold increase in the ALP enzyme is considered characteristic of a cholestatic injury [32,33]. Regarding liver function and organ damage, no alterations were observed in the present study despite an increase of 1.20 in ALP above the upper limit of normal values after treatment with CP-F8 and an increase in ALT of 1.57 and 1.15 in the control and vehicle control groups respectively, as these are below the limits reported for hepatocellular or cholestatic injury.

Similarly, the analysis of this type of enzyme has limitations since it may be altered by damage to different organs, such as bone and muscles, without being related to drug-induced liver injury [34]. Histological examination or biopsy usually shows marked necrosis and inflammation [33]. However, the histological sections obtained from our specimens support the results of the enzymatic analysis, showing no hepatocellular damage, necrosis, or fibrosis for the three groups used.

## 4. Materials and Methods

### 4.1. Plant Material

The plant material was collected in Guanajuato city, Guanajuato, Mexico (102°5′49.2″ W to 99°40′16.68″ W 19°54′46.08″ N to 21°50′21.84″ N). The specimens were identified by Sc.D. María Magdalena Salinas Rodríguez at the Herbarium of the Faculty of Biological Sciences at Universidad Autónoma de Nuevo León, Nuevo León, Mexico, with voucher number 25588.

### 4.2. Preparation of Extracts

One hundred grams of ground and dried plant material were subjected to extraction with 500 mL of methanol (CTR Scientific, Nuevo León, Mexico) for 48 h in a Soxhlet extractor to obtain the crude methanol extract (LmM). Partitions were obtained from the resulting extract using the same apparatus with *n*-hexane, chloroform, and methanol (CTR Scientific) for 48 h each to obtain the *n*-hexane (LmPH), chloroform (LmPC), and methanol (LmPM) partitions. The extract and partitions were filtered through Whatman No. 1 filter paper (Merck, Darmstadt, Germany) and subsequently concentrated to dryness using a Büchi R-3000 rotary evaporator (Büchi Labortechnik, Flawil, Switzerland). The percentage extraction yield was calculated using the following formula:

$$\%\;Yield=\left(\frac{Final\;extract\;weight}{Initial\;extract\;weight}\right)\times 100$$

### 4.3. Fractionation

The fractionation of 500 mg of chloroform partition was performed on a chromatographic column with 25 g of silica gel normal phase from 0.063 mm to 200 mm (Merck, Darmstadt, Germany) suspended in chloroform as packing solvent [10], after which 20 mL elutions were achieved in gradients of increasing polarity, using chloroform, chloroform-ethyl acetate, ethyl acetate, ethyl acetate-methanol, and methanol. Five milliliters of fractions were then collected at elution times of 15 drops/min.

### 4.4. Phytochemical Analysis

The phytochemical profile of CP-F8 was determined by qualitative analysis to identify the presence of various compounds and functional groups. This analysis was performed in a single repetition in test tubes, using a solution of the CP-F8 fraction dissolved in methanol at a concentration of 1 mg/mL. Methanol dilution solvent was used as a negative control. The tests were interpreted visually, with a positive test being one that showed physical or color changes and a negative test being one that showed no changes according to the tests described below.

The concentrated hydrochloric acid (HCl) was used to detect xanthophylls, with 0.2 mL to 0.5 mL of the fraction solution being added. A change in color to blue-green is considered positive [35]. Baeyer’s test was used to detect unsaturated carbon–carbon bonds. This was achieved by adding 1% potassium permanganate (KMnO_4_) to the CP-F8 solution in a 1:2 ratio, stirring, and allowing it to stand for one minute. A brown color change or the appearance of a precipitate is considered a positive result [36]. The presence of carbohydrates was determined through the implementation of Molisch’s test, a method that entails the addition of 1 mL of Molisch’s reagent to 2 mL of the methanolic solution. Then, 1 mL of sulfuric acid was carefully added to the side of the test tube. The presence of carbohydrates in the extract will be observed when a violet ring forms at the interface of the solution. The assay for the detection of phenolic compounds employed the 5% ferric chloride (FeCl_3_) reagent, with the addition of 1–2 drops of the reagent to 1 mL of the CP-F8 solution. The presence of phenolic hydroxyl groups is indicated by a change in color to blue, green, purple, or red. The Shinoda’s test was employed for the detection of phenolic compounds such as flavonoids. In this test, a small piece of Mg was added to 1 mL of the partition, followed by 10 drops of concentrated hydrochloric acid. The presence of flavonoids is indicated by a color change in the solution, which typically turns pink or red. For the analysis of steroids and triterpenes, the Liebermann–Burchard test was employed, wherein 2 mL of the reagent was added to 1 mL of the partition. A color change to pink, blue, or green is considered a positive test result. The presence of coumarins was detected using 10% sodium hydroxide, which was added in a 1:2 ratio to the fraction sample. A positive test result is indicated by a yellow color change. Lactone groups were detected using the Baljet test, adding five drops of Baljet reagent to 1 mL of the partition. A color change to orange or reddish-orange signifies a positive test result. Finally, for the detection of alkaloids, the Dragendorff’s test was used, with 1 mL of the reagent added to 2 mL of the solution. The presence of a brown precipitate or a yellow color change indicates a positive test result [37,38,39].

### 4.5. Cell Lines

In this study we used murine lymphoma L5178Y-R (ATCC CRL-1722 TM) cells (ATCC; Manassas, VA, USA). Peripheral blood mononuclear cells (PBMC) were obtained from the blood of healthy volunteer donors, using Ficoll-Paque PLUS (GE Healthcare, Piscataway, NJ, USA). L5178Y-R cells were maintained in RPMI-1640 culture medium (Merck) supplemented with 10% FBS and 1% antibiotic–antifungal solution (Sigma-Aldrich, St. Louis, MO, USA). VERO cells were maintained in Dulbecco’s Modified Eagle Medium (DMEM) (Thermo Fisher Scientific Inc., Waltham, MA, USA) supplemented with 10% FBS and 1% antibiotic–antifungal solution (Sigma-Aldrich). All cells were cultured at 37 °C in an atmosphere with 5% CO_2_ in 95% air.

### 4.6. Cell Viability Assay

The L5178Y-R cell line was cultured at a density of 1 × 10^4^ cells/well and the VERO and PBMC at 1 × 10^5^ cells/well for 24 h and treated with 1:2 serial dilutions of *L. marginatus* extracts and fractions from 250 mg/mL in a final volume of 200 μL for 48 h at 37 °C in a 5% CO_2_ atmosphere. We used 0.5 μg/mL of vincristine sulfate (Hospira, Warwickshire, UK) as a positive control and culture medium alone as a negative control. All assays were performed in round-bottomed 96-well microplates (Corning; Corning, NY, USA). Cell viability was assessed by the colorimetric reduction assay with 3-[4,5-dimethylthiazol-2-yl]-2,5-diphenyltetrazolium bromide (Merck) by adding 15 μL of MTT/well (5 mg/mL final concentration) and incubating at 37 °C for 4 h. Formazan crystals were dissolved with dimethyl sulfoxide (Sigma-Aldrich) and optical densities (OD) were measured at 570 nm, using a MULTISKAN GO microplate reader (Thermo Fisher Scientific) [40]. The percentage of viability inhibition was calculated using the following formula:

$$\%\;Inhibition=\left[\left(\frac{{OD}_{570}\;treatment}{{OD}_{570}\;negative\;control}\right)\times 100\right]$$

Selectivity indices (SIs) were obtained by dividing the IC_50_ of PBMC by that of the respective tumor cell line. Any sample with an SI value > 10 was considered of high selectivity [14].

### 4.7. Hemolysis Assay

The hemolytic activity of the extracts was determined using the method described above [15]. Twenty milliliters of blood were obtained from a healthy volunteer in tubes containing EDTA. Red blood cells were washed three times with phosphate-buffered saline (PBS) at pH 7.2, and erythrocytes were prepared as a 5% suspension in sterile PBS. Subsequently, the concentrations of the extracts to be evaluated (200 μg/mL, 400 μg/mL, 600 μg/mL, 800 μg/mL, and 1000 μg/mL) and the erythrocyte suspension were added in triplicate to 2 mL tubes. Distilled water was used as positive control, and PBS as negative control. Samples were then incubated at 37 °C for 30 min and centrifuged at 4 °C for 5 min at 13,000 rpm. The percentage of hemolysis was calculated using the following formula:

$$\%\;Hemolysis=\left[\left(\frac{{OD}_{540}\;treatment}{{OD}_{540}\;positive\;control}\right)\times 100\right]$$

### 4.8. DPPH Antioxidant Activity

An antioxidant activity assay was performed using the 2,2-diphenyl-1-picrylhydrazyl (DPPH) method [41]. Methanol was used as a negative control, and ascorbic acid at concentrations of 1.95 μg/mL to 250 μg/mL was used as positive control. We then placed 100 μL of extract at different concentrations and 100 μL of 120 μM DPPH in a microplate and then incubated for 30 min at room temperature in darkness. ODs were then measured at 517 nm, using a microplate reader, and the percentage of DPPH radical inhibition was calculated using the following formula:

$$\%\;DPPH\;inhibition=\left[\left(\frac{{OD}_{517}\;control-{OD}_{517}\;sample}{{OD}_{517}\;control}\right)\times 100\right]$$

### 4.9. Animals

We used groups of four 12- to 14-week-old BALC/c mice, approximately weighing 36 g. They were provided by the Animal Facility of the Immunology and Virology Laboratory of the School of Biological Sciences at Autonomous University of Nuevo Leon UANL), Mexico. They were kept in ventilated cages with access to food and water *ad libitum*, under a 12 h light/dark cycle in a stress-free, pest-free, and pathogen-free environment at a temperature of 22 °C and 45% relative humidity. Established guidelines for the care and welfare of animals in cancer research were followed [42]. A clinical score was used to assess body weight, fur condition, posture, and social activity to determine the animals’ evaluation criteria [43].

### 4.10. Extracts Maximum Tolerated Dose and Hepatotoxicity

The maximum tolerated dose test was performed in accordance with the recommendations published by the Organization for Economic Cooperation and Development (OECD) [43]. Extracts were resuspended in 200 µL of a PEG-300 vehicle (Sigma-Aldrich). Treatment was intraperitoneally administered as a single dose of 100 mg/kg to each mouse in the group, in addition to maintaining a vehicle group and a control group. These mice were closely observed for any signs of toxicity, including mortality, lacrimation, physical or behavioral abnormalities, ruffled fur, and other symptoms. Monitoring was carefully performed at 30 min intervals for four hours and daily for 14 consecutive days [44]. Humane euthanasia was performed if there was a weight loss ≥ 20%. For cardiac puncture, a dose of 25 to 40 mg/kg of sodium pentobarbital (Aranda Animal Health, Querétaro, Mexico) was intraperitoneally administered as an anesthetic, followed by obtaining a blood sample and euthanasia. Blood samples were centrifuged in BD microtainer tubes (Franklin Lakes, NJ, USA) with serum separator gel at 3000 rpm for five minutes to obtain serum for liver function testing. Livers from the specimens were collected and stored in 10% formalin for fixation and subsequent histological analysis [45].

### 4.11. Statistical Analysis

Statistical analyses were performed using GraphPad Prism 9.0.1 software (GraphPad Software Inc., San Diego, CA, USA). Cytotoxicity results were expressed as the mean ± SEM of triplicate determinations from three independent experiments. Survival data were processed using Kaplan–Meier survival analysis, followed by a record-rank test with *p* < 0.05. One-way ANOVA was performed to determine significant differences. Tukey’s or Dunnett’s post hoc tests were used as required. IC_50_ values were calculated using the Probit test.

## 5. Conclusions

Plants have long been an important source of secondary metabolites for new cancer therapies. This study demonstrated that the CP-F8 fraction, which was isolated from the chloroform extract of *L. marginatus*, exhibits desirable anti-cancer activity in vitro against the murine lymphoma cell line L-5178Y-R. Qualitative phytochemical analysis revealed the presence of unsaturated carbon–carbon bonds, phenolic hydroxyl groups, and steroids in our fraction. According to the literature, these functional groups may be due to metabolites such as phenolic acids and phytosteroids, which have anti-cancer and antioxidant activities, as observed in this study. The CP-F8 fraction’s safety was verified by demonstrating no negative effects on healthy cells. It proved to be highly selective and did not generate hemolysis in in vitro assays. Similarly, no liver damage was observed in an in vivo model. These results allow us to categorize CP-F8 as a candidate for purification. However, further chromatographic and spectroscopic studies are necessary to completely characterize the compounds present and determine their antitumor activity in an in vivo model.

## Figures and Tables

**Table 1:** Extraction yield and IC_50_ of *L. marginatus* crude extract and partitions.

Extract	% Yield	L5178Y-R (μg/mL) *	PBMC (μg/mL) *	SI	5% Erythrocytes (μg/mL) *
LM-CME	15.22 **	9.09 ± 2.140	>3000	330.03	>1000
LM-HP	16.58 ***	6.74 ± 1.3	>3000	445.1	<200
LM-CP	18.00 ***	7.93 ± 2.972	>3000	378.31	>1000
LM-MP	50.23 ***	45.38 ± 2.549	>3000	66.1	>1000

* IC_50_ was obtained using nonlinear Probit regression. Data are mean ± SEM of triplicate determinations from three independent experiments. Vincristine was used as a positive control. ** Percentage of 1275 g of dry plant material; *** Percentage of 154 g of LM-CME extract.

**Table 2:** IC_50_ of chloroform partitioning fractions.

Fraction	% Yield **	L5178Y-R (μg/mL) *	PBMC (μg/mL) *	SI	5% Erythrocytes (μg/mL) *
CP-F1	1.26	66.94 ± 1.050	15.22	0.23	ND
CP-F2	7.55	21.89 ± 1.063	777.7	35.53	ND
CP-F3	1.55	25.56 ± 1.038	552.1	21.60	ND
CP-F4	1.17	24.15 ± 1.040	302.8	12.54	ND
CP-F5	5.92	29.16 ± 1.074	105.2	3.61	ND
CP-F6	7.64	31.14 ± 1.068	238.1	7.65	ND
CP-F7	11.68	21.23 ± 1.116	45.07	2.12	ND
CP-F8	18.05	11.20 ± 1.093	3968	354.29	>1000

* IC_50_ was obtained using nonlinear Probit regression. Data are mean ± SEM of triplicate determinations from three independent experiments. Vincristine was used as a positive control. ** Yield of 10 g of LM-CP; ND: not determined; CP-F8 was selected only for its yield, biological activity, and selectivity.

**Table 3:** Phytochemical analysis of CP-F8.

Assay	Metabolite/Functional Group	Test Result	Interpretation
Concentrated hydrochloric acid	Xanthophyll	There was no change in coloration to greenish blue	Negative
Baeyer	Unsaturated carbon–carbon bond	The formation of a precipitation was observed	Positive
Molisch	Carbohydrates	No violet ring formed at the interface	Negative
Shinoda	Flavonoids	No pink or red color change observed	Negative
Dragendorff	Alkaloids	There was no yellow color change or presence of brown precipitate	Negative
Ferric chloride	Phenols	A green color change was observed	Positive
Liebermann-Burchard	Steroids and Triterpenes	A green color change was observed	Positive
Sodium hydroxide (10%)	Coumarins	There was no change in coloration to yellow	Negative
Baljet	Lactones	No change in color to orange or reddish orange was observed	Negative

**Table 4:** Blood biochemical profile of Balb/c mice.

Parameters	Untreated Control	Vehicle	CP-F8 (100 mg/kg)	Reference Value [11]
Glucose (mg/dL)	96 (0.41) *	164 (0.70)	186 (0.79)	21.4–232.6
Total protein (g/dL)	5.2 (0.89)	5.1 (0.87)	4.9 (0.84)	4.60–5.80
Albumin (g/dL)	2.9 (0.69)	2.7 (0.64)	2.8 (0.66)	2.70–4.20
ALP (U/L)	159 (0.77)	194 (0.94)	246 (1.20)	46.0–204.8
Aspartate aminotransferase (AST) (U/L)	427 (0.65)	305 (0.46)	265 (0.40)	39.30–655.80
ALT (U/L)	316 (1.57)	232 (1.15)	176 (0.87)	18.20–200.20

* Values in parentheses represent the fold increase/decrease in the upper normal limit. U/L: units per liter; g/dL: grams per deciliter; mg/dL: milligrams per deciliter.

## Data Availability

The original contributions presented in this study are included in the article. Further inquiries can be directed to the corresponding authors.
